# Hierarchical Shared Control of Cane-Type Walking-Aid Robot

**DOI:** 10.1155/2017/8932938

**Published:** 2017-08-13

**Authors:** Chunjing Tao, Qingyang Yan, Yitong Li

**Affiliations:** ^1^Beijing Key Laboratory of Rehabilitation Technical Aids for Old-Age Disability, The National Research Centre for Rehabilitation Technical Aids, Beijing 100176, China; ^2^Key Laboratory of Ministry of Education for Image Processing and Intelligent Control, School of Automation, Huazhong University of Science and Technology, Wuhan 430074, China; ^3^The 28th Research Institute of China Electronics Technology Group Corporation, Nanjing 210007, China

## Abstract

A hierarchical shared-control method of the walking-aid robot for both human motion intention recognition and the obstacle emergency-avoidance method based on artificial potential field (APF) is proposed in this paper. The human motion intention is obtained from the interaction force measurements of the sensory system composed of 4 force-sensing registers (FSR) and a torque sensor. Meanwhile, a laser-range finder (LRF) forward is applied to detect the obstacles and try to guide the operator based on the repulsion force calculated by artificial potential field. An obstacle emergency-avoidance method which comprises different control strategies is also assumed according to the different states of obstacles or emergency cases. To ensure the user's safety, the hierarchical shared-control method combines the intention recognition method with the obstacle emergency-avoidance method based on the distance between the walking-aid robot and the obstacles. At last, experiments validate the effectiveness of the proposed hierarchical shared-control method.

## 1. Introduction

With the development of society, the aging of population has caused more and more important social problems [1]. The elderly are faced with the problems such as weakening of physical ability and visual deterioration as they grow older. Thus, the elderly need to improve their walking ability to cope with their basic needs of daily life indeed. Opportunely, many researchers have paid attention to the applications of the robot and medical technology in recent years [2–10]. They manufactured many intelligent rehabilitation aids (e.g., walking-aid robots) to help the elderly to gain the ability of normal walking and developed a series of achievements, such as Kawamoto et al. invented “HAL” [5]. A wearable Power-Assist Locomotor (WPAL) was invented for the lower limb [6]. Kikuchi et al. [7] proposed an intelligently controllable walker (i-walker). Hirata et al. invented a passive intelligent walker called “RT-Walker” [9]. Wakita et al. [10] also invented a cane-type walking-aid robot “i-cane” to help elderly walk and rehabilitate.

In addition, another hot point of the robotics industry is the obstacle avoidance. These investigations generally need various sensors such as ultrasonic sensors, laser sensors, and cameras. Combined with some specific algorithms, the autonomous navigation and obstacle avoidance function of robots can be achieved. Currently, a great deal of researches have been published [11–14]. References [15, 16] proposed the obstacle detection and avoidance methods for the robot with a camera. However, the process of the images will make the computation more complicated, which will also cost more time and is not suitable for walking-aid robots.

Based on aforementioned researches, it can be found that the walking-aid robots have good human-machine interaction interfaces and there are many human motion intention recognition methods which can fully consider the human's subjective intention [17–24]. The admittance control performs well in using walking-aid robots [10]. However, these walking-aid robots usually cannot recognize and rectify the operator's unreasonable or incorrect intentions, which may cause some safety hazard. In comparison with the walking-aid robots, the obstacle avoidance robots have the function of path planning and can reach the target point safely. Regretfully, the obstacle avoidance robots only have the single function and lack of the human-machine interface.

Considering the advantages and disadvantages of the robot control and the human control, many investigators combined advantages of these two control methods and proposed the concept of shared control. The shared control is defined that a system can share its controller with one or more human beings and one or multiple robotic controllers [25]. In the field of shared control, many researchers gained quite a few achievements [26–30]. Overall, the research on the shared-control robot is still in its infancy. References [31, 32] proposed a shared-control method for the wheel robot with detection of the human intention through the EMG. But devices for obtaining the bioelectricity signals with pins cannot offer comfortable experiences. Meanwhile, those devices are expensive and not convenient to use in daily life without professional staffs around.

In this study, a convenient and cost-effective hierarchical shared-control method of the walking-aid robot based on human motion intention recognition and obstacle emergency-avoidance methods is presented for solving the situation if there are obstacles during the normal walking. It can save effort during obstacle avoidance and keeps part of the operator's original walking intention. The walking-aid robot is introduced in Section 2. The hierarchical shared-control method which considers both the walking-aid and the obstacle avoidance functions in the walking-aid robot is introduced in Section 3 in detail. In particular, the intention recognition algorithms can make the walking-aid robot thoroughly consider the operator's subjective intention and enhance the quality of human-computer interaction. Also, the artificial potential field method used in this paper can plan the path of walking-aid robot to avoid risks caused by the operators' unreasonable intentions. Furthermore, different control strategies are assumed according to the distance between the walking-aid robot and the obstacles.  Section 4 talks about the experiments and analysis. Conclusions are made in Section 5. Finally, experiments are conducted in the real environment which proves the effectiveness of the proposed shared-control algorithm.

## 2. Walking-Aid Robot System

### 2.1. Architectural Structure of the Walking-Aid Robot

**Figure 1:**
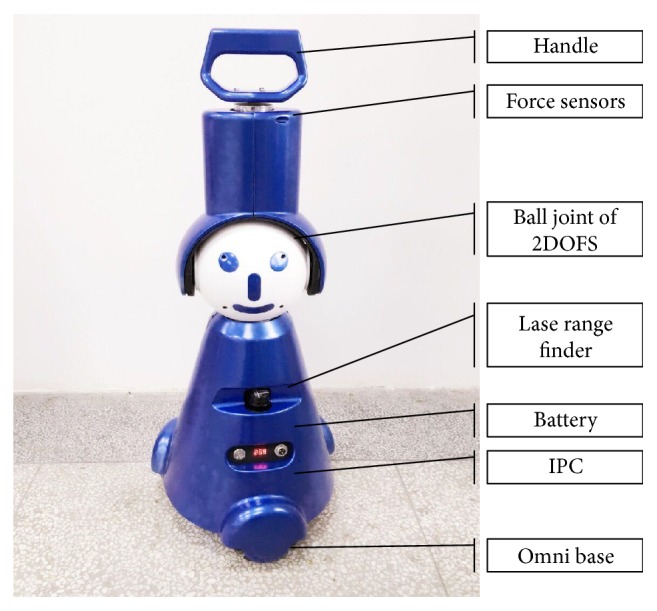
The walking-aid robot.

In this work, the walking-aid robot system is composed of a set of solid support structures, an omni-directional platform, an industrial personal computer (IPC), a laser sensor, a force-sensing device with FSR, and a torque sensor. The actual photo of the walking-aid robot is shown in Figure 1. The omni-directional platform consists of three omni-directional mecanum wheels driven by DC motor. The laser sensor is adopted to detect obstacles around. The operation principle of the force-sensing device will be stated in the following section.

**Figure 2:**
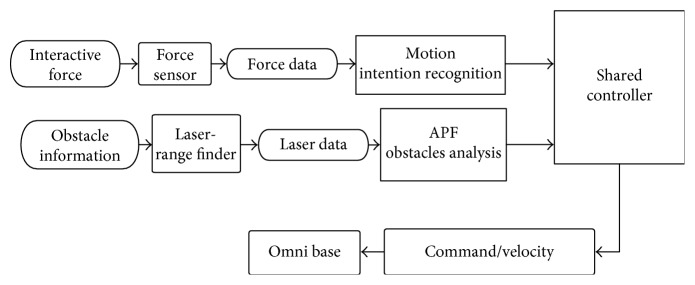
Architecture of control system applied in the walking-aid robot.

Figure 2 shows the control flowchart of the walking-aid robot. In this system, the interactive force from the operator collected by the laser sensor can be transmitted to the IPC. IPC can send control commands based on the collected data to the platform to control the movements of the walking-aid robot.

### 2.2. The Force-Sensing Device in Walking-Aid Robot

**Figure 3:**
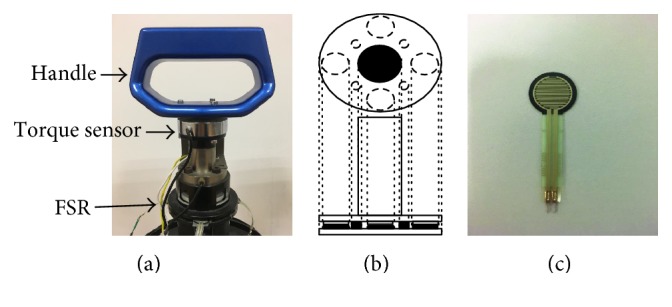
Force sensors.

Figure 3 shows the force-sensing device in the walking-aid robot in detail.  Figure 3(a) shows the structure of the force-sensing device including the handle, the torque sensor, and the FSR for four directions.  Figure 3(b) shows the distribution of the four FSR sensors which are pasted to the four sides of the metal rod of the handle, thus making the FSR sensors and the torque sensor precisely detect the magnitude and direction of the force from the operator.  Figure 3(c) shows the FSR sensor, which is a one-dimensional variable-resistance pressure sensor. Connecting these FSR sensors and the torque force sensor to the signal conditioning circuits, the force from the operator shall be obtained as the intention force.

### 2.3. The Establishment of Coordinate System

**Figure 4:**
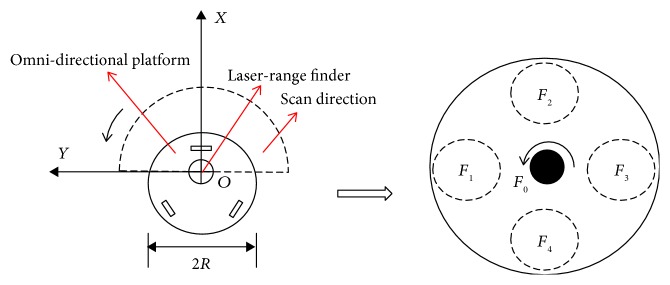
Top view of the walking-aid robot system.

Figure 4 shows the top view of the walking-aid robot architecture. Because the shared-control algorithm is just based on the human motion intention recognition and the local obstructions around the walking-aid robot, a global coordinate system is not necessary in this study. But the coordinate systems of the omni-directional platform, the force-sensing device, and the laser sensor need to be established. In order to simplify the calculations, the coordinate systems of the omni-directional platform, the force-sensing device, and the laser sensor are unified with a fixed local coordinate system *XOY*. In the coordinate system *XOY*, the front direction of the walking-aid robot is set as the positive *x*-axis and the left anterior side is set as the positive *y*-axis. Because the FSR sensors are one-dimensional pressure sensors, the data obtained by the force-sensing device is the component of the operator's force in *X* or *Y* direction. We assume that the force along the positive *y*-axis is *F*_1_. Along the clockwise direction, the force detected by the torque sensor is *F*_0_. The forces towards four directions acquired by the FSR sensors are *F*_1_, *F*_2_, *F*_3_,  and *F*_4_, respectively. The laser sensor scans the front obstacles in the counterclockwise direction continuously to acquire the environment information. In the local coordinate system *XOY*, the positive *X* direction is set as the initial angle 0°, and the angle increases along the counterclockwise direction. In this case, the scanning range of the laser sensor is [−90°, 90°].

## 3. Shared-Control Algorithm

### 3.1. The Algorithm for Admittance Control

From the force-sensing device described in 2.2 and the forces *F*_0_ − *F*_4_in 2.3, we can get the components of the operator's intention force along the *x*-axis and *y*-axis and the rotate direction, respectively. And the operator's intentions are *F*_*X*_, *F*_*Y*_,  and *M*_*Z*_. Thus, the force components can be obtained by

(1) $$\begin{array}{c} \begin{array}{l} F_{X}=F_{1}-F_{3}\\ F_{Y}=F_{2}-F_{4}\\ M_{Z}=F_{0}R. \end{array} \end{array}$$

In this study, the operator's intention forces are represented by a five-dimensional vector and the operator's intentions are represented by a three-dimensional vector. Then, the intention forces and operator's intentions can be expressed as

(2) $$\begin{array}{c} F_{S}={\left[\;F_{0}\;F_{1}\;F_{2}\;F_{3}\;F_{4}\;\right]}^{T},\\ F_{I}={\left[\begin{array}{ccc} F_{X} & F_{Y} & M_{Z} \end{array}\right]}^{T}. \end{array}$$

*F*_*X*_ is the intention force along the direction of the *x*-axis. *F*_*Y*_ is the intention force along the direction of the *y*-axis, and *M*_*X*_ is the torque exerted on the walking-aid robots. The positive direction of the torque is counterclockwise direction in this article. Then, the intention forces can be rewritten into

(3) $$\begin{array}{c} F_{I}=E_{IS}F_{S}. \end{array}$$

*E*_IS_ is a transformation matrix which is defined by

(4) $$\begin{array}{c} E_{IS}=\left[\begin{array}{lllll} 0 & 0 & 1 & 0 & -1\\ 0 & 1 & 0 & -1 & 0\\ R & 0 & 0 & 0 & 0 \end{array}\right]. \end{array}$$

*R* is half the width of the walking-aid robot in Figure 4.

Once the vector of the operator's intention force *F*_I_ is obtained, the vector *F*_I_ can be converted into a velocity vector *V* through the open-loop controlled admittance algorithm. According to the admittance control algorithm, the transfer function can be written as

(5) $$\begin{array}{c} \frac{V\left(s\right)}{F_{I}\left(s\right)}=\frac{k}{\tau s+1}. \end{array}$$

*k* is the proportional coefficient. Further, (5) can be taken into the form of continuous time domain model:

(6) $$\begin{array}{c} \tau \frac{dV\left(t\right)}{dt}+V\left(t\right)={kF}_{I}\left(t\right). \end{array}$$

Discretizing (6), we have

(7) $$\begin{array}{c} \tau \frac{V\left(i\right)-V\left(i-1\right)}{T}+V\left(i\right)={kF}_{I}\left(i\right). \end{array}$$

Finally, (7) can be rewritten in a simplified form as discretizing (6), we have

(8) $$\begin{array}{c} V\left(i\right)=k\frac{T}{T+\tau }F_{I}\left(i\right)+\frac{\tau }{T+\tau }V\left(i-1\right). \end{array}$$

### 3.2. The Artificial Potential Field Approaches

The repulsion force based on the artificial potential field approaches is given by the formula

(9) $$\begin{array}{c} F_{ri}=K{\left(r-R_{0}\right)}^{-n}. \end{array}$$

*K*, *R*_0_, and *n* are constant. The constant *n* is a positive integer, and *r* is the distance between an obstacle and the walking-aid robot. *F*_ri_ represents the repulsion force. Researchers have proposed an effective obstacle recognition algorithm to calculate the repulsion force in [33]. The linked list of obstacles which reflects the information of the around obstructions can be obtained by the simplified obstacle recognition algorithm.

**Figure 5:**
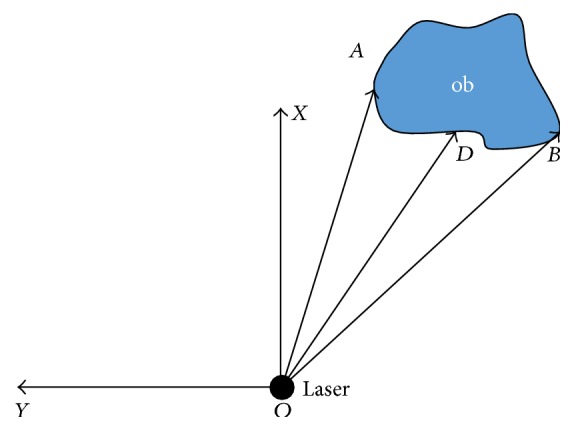
The structure of the obstacle (ob).

Each obstacle in the environment could be expressed in a six-tuple structure [*rEdge*, *rAngle*, *lEdge*, *lAngle*, *Force*, and *Angle*], as shown in Figure 5.

The laser sensor locates at the point *O*. The obstacle is ob. *rEdge* represents the distance |*OB*| between the obstruction's right edge and the laser sensor. *rAngle* is the angle between the *x*-axis and the line which connects the right edge of obstruction and the laser sensor. Similarly, *lEdge* and *lAngle* represent the distance |*OA*| and the angle ∠*AOX*, respectively. *Force* is the magnitude of the repulsion force, and *Angle* is the angle between the positive *x*-axis and the direction of repulsion force. For each obstacle in this study, the direction of repulsion force is defined by the angle bisector *OD* of the angle ∠*AOB* which is between the edge of the obstacle and the detection line of laser sensor. Substituting *r* = |*OD*| into (9), the repulsion force caused by the single obstacle ob can be obtained as

(10) $$\begin{array}{c} F_{ob}=K{\left(\left|OD\right|-R_{0}\right)}^{-n}. \end{array}$$

**Figure 6:**
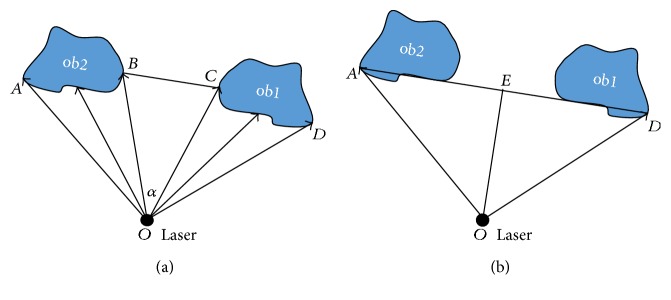
Diagram of the merging obstacles ob1 and ob2.

It should be noted that the gap between two obstacles may be so small that the walking-aid robot cannot pass through the gap safely. Since the repulsion force caused by an obstacle is only determined by the relative distance between the obstacle and the walking-aid robot, the accumulative repulsive force caused by the group of obstacles in the same location may be greater than the operator's intention force. In this case, the accumulative repulsion force will hinder the motion of the walking-aid robot. To avoid this case, the obstacles with short distance should be merged.  Figure 6() gives a diagram of the merging obstacles.

For the two adjacent obstacles ob1 and ob2 as shown in Figure 6(a), the value of the angle ∠*BOC* can be easily obtained based on the given six-tuple structure. According to the law of cosines, the distance |*BC*| between ob1 and ob2 is found as

(11) $$\begin{array}{c} \;|\;BC\;|\;=\sqrt{{\left|OB\right|}^{2}+{\left|OC\right|}^{2}-2\left|OB\right|\left|OC\right|\cos\alpha }. \end{array}$$

If the distance is smaller than the size of the walking-aid robot, the two obstacles need to be merged, as shown in Figure 6(b). The merged margin of the two obstacles is determined by the right edge of obstacle ob1 and the left edge of obstacle ob2. Then, the distance of the angle bisector is obtained. Based on (9), the repulsion force caused by the merged obstacle can be calculated as

(12) $$\begin{array}{c} F_{ob}=K{\left(\left|OE\right|-R_{0}\right)}^{-n}. \end{array}$$

The distance |*OE*| cannot be directly determined by the laser sensor. The result is that the light beam from the laser sensor (the line extension cord) might just cross the gap between two obstacles. If this happens, the laser sensor cannot detect obstacles and the repulsion force cannot be obtained. The information of merged obstacles is stored in the list, and the resultant of repulsion forces is given by

(13) $$\begin{array}{c} F_{r}=\overset{n}{\underset{i=0}{\sum }}F_{ob\left(i\right)}. \end{array}$$

In practical application, the components of the repulsion forces *F*_ob_ along the *X* and *Y* direction are calculated, respectively, in order to facilitate the fusion with the intention force. The positive and negative direction of the repulsion forces is consistent with the *x*-axis and the *y*-axis, respectively. The corresponding components can be obtained as

(14) $$\begin{array}{c} F_{obX}=-\overset{n}{\underset{i=0}{\sum }}\left|F_{ob\left(i\right)}\right|\cos\theta_{i},\\ F_{obY}=-\overset{n}{\underset{i=0}{\sum }}\left|F_{ob\left(i\right)}\right|\sin\theta_{i}. \end{array}$$

### 3.3. The Algorithm of Obstacle Emergency Avoidance

**Figure 7:**
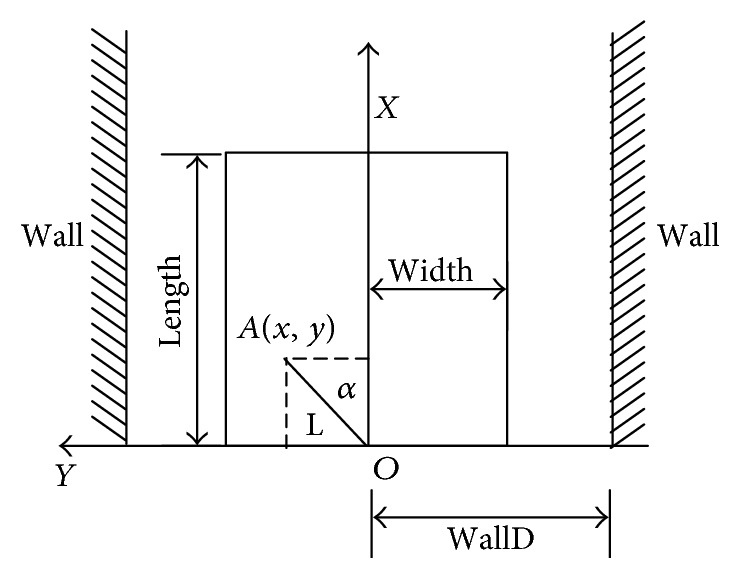
The obstacle emergency-avoidance region.

The short distance between the walking-aid robot and the obstacle may cause the collision. Thus, the algorithm of obstacle emergency avoidance must be taken to ensure the operator's safety and the safety of the robot. Considering the features of the walking-aid robot, an obstacle avoidance strategy is proposed in this study. When the walking-aid robot moves, the obstacle emergency-avoidance region is firstly determined based on the strategy. If the component of the intention force along the *x*-axis is negative when there are obstacles in the region, the walking-aid robot will move back to avoid obstacles. If not, the walking-aid robot will move in the lateral way to avoid obstacles. In this study, the selected obstacle emergency-avoidance region is a rectangle while the laser sensor locates at point *O* as shown in Figure 7(). If a scanning point of the laser sensor is detected in the rectangular area, it means that there are obstacles in the area. The rectangle condition is described as follows:

(15) $$\begin{array}{c} \begin{array}{l} \left|L\cos\alpha \right|<length\\ \left|L\sin\alpha \right|<width. \end{array} \end{array}$$

The point *A* (*x*, *y*) is the detection point of the laser sensor and |*OA*| = *L*. When (15) holds, the rectangle condition is true.

In Figure 7, the “WallD” represents the distance between the walking-aid robot and the lateral wall.

**Figure 8:**
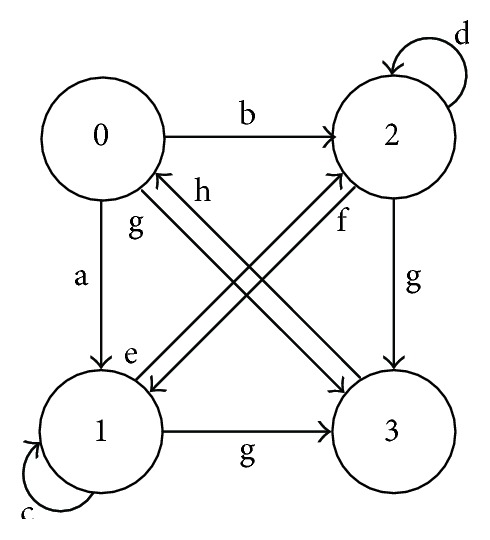
The state transition diagram of obstacle emergency-avoidance method.

As the first step, the algorithm can make the robot move parallel along the positive or negative direction of the *y*-axis to avoid obstacles. For the selection of the robot movement direction, a voting algorithm is used. The robot counts the numbers of the scanning points which satisfy the rectangle condition (15) within each side of *x*-axis. Then, the side where fewer scanning points satisfying the rectangle condition (15) exist is considered as the enough space for the obstacle avoidance. Thus, it will move to the side where fewer scanning points satisfying the rectangle condition (15) exist. Based on the voting algorithm, the robot will move to the side where fewer scanning points exist. The initial state of the walking-aid robot is also determined by using the voting algorithm. Above all, the proposed obstacle emergency-avoidance method can be described by the state transition diagram, as shown in Figure 8(). The numbers 0–3 represent the states of the walking-aid robot as shown in Table 1, and the letters “a–h” represent the state-transition conditions in Table 2.

During the obstacle avoidance, the robot detects the distance “WallD” all the time. Once the distance is less than the safe distance, the robot can change its state of motion immediately. The walking-aid robot can convert among various motion states when there are obstacles in the obstacle avoidance region.

### 3.4. The Hierarchical Shared-Control Algorithm

From the above descriptions, the admittance control algorithm proposed in Section 3.1 can effectively obtain the corresponding velocity of the robot based on the operator's intention force. The admittance control algorithm can also make the operator control smoothly. In Section 2.2, the artificial potential field method synthetically considers the obstacles around the robot and the operator's intention force, which is conducive to make the robot move towards a reasonable direction. The obstacle emergency-avoidance method proposed in Section 3.3 can fully guarantee the safety of the robot and avoid obstacles in the mean time.

**Figure 9:**
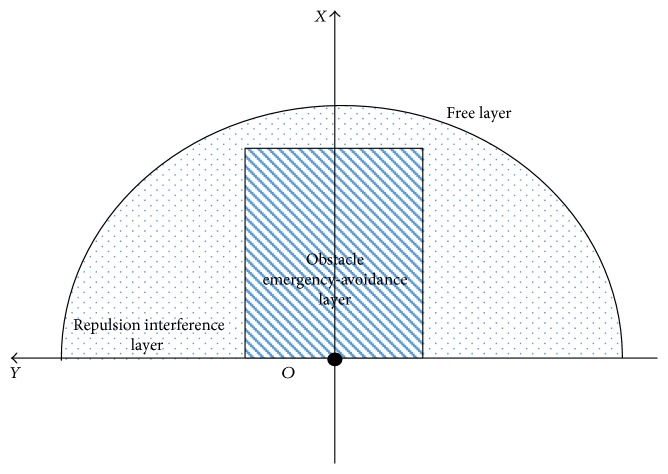
The hierarchical layers of the shared-control algorithm.

Integrating above three algorithms, the shared-control algorithm can be designed easily. According to different distances between the robot and the obstacles in the hierarchical way as shown in Figure 9(), the shared-control algorithm can use different control algorithms to control the robot, as shown in Figure 0() and Hierarchical Shared-Control Algorithm.

In the control system, the laser sensor is set as the center and the outermost is set as the **Free Layer**. Without obstacles in the semicircular region shown in Figure 9(), the robot is in fully compliance with the operator's intention. The region between the semicircular region and the rectangular region is defined as the **Repulsion Interference Layer**. If any obstacle is detected in this layer, the repulsion force calculated by the artificial potential field method and the operator's intention force control the movement of the robot in the same time. The region within the rectangular region is defined as the **Obstacle Emergency-Avoidance Layer**. If any obstacle is detected in this layer, the robot will only receive the backward movement instruction from the operator; otherwise, it will follow the algorithm of obstacle emergency-avoidance proposed in Section 3.3 to avoid obstacles.

## 4. Experiment and Analysis

In this work, the experiments are conducted in the specific indoor environments depicted in Figure 1(). In the experiments, constant *n* in (9) is selected as 2 to ensure and the repulsion force intensively depends on the distance between the obstacles and the walking-aid robot. Meanwhile, the value of repulsion force should match with the magnitude of the intention force which the sensors measure. According to the AD conversion result of the IPC, the valid range of the intention force is approximately 1–25 N. When the distance *r* = 1.5 m, the obstacle is very far from the walking-aid robot and its repulsion force is so small that cannot affect the movements of the walking-aid robot. At this time, the repulsion force *F*_ri_ = 1 N. When the distance *r* = 0.4 m, the obstacle is very close to the walking-aid robot. At this time, the repulsion force shall roughly be equivalent to the maximum intention force, namely, *F*_ri_ = 25 N. Substituting these constants into (9), we can obtain the parameters: *K* = 1.313, *R*_0_ = 0.354. In the experiment, the walking-aid robot is pushed forward. Figure 1() shows the movement trajectory of the walking-aid robot.

The experiment is conducted with a 24-year-old student and a 23-year-old student. The experimental results are shown in Figures 2() and 3(). In the figures, the horizontal axis is a time coordinate. *HFx* and *HFy* represent the intention forces along the *x*-axis and *y*-axis, respectively. *Rx* and *Ry* represent the repulsion forces along the *x*-axis and *y*-axis, respectively. *Vx* and *Vy* represent the velocities of the walking-aid robot along the *x*-axis and *y*-axis, respectively.

From the above three figures, the experiments can be divided into three stages as below:

*Stage 1*. At the beginning, the walking-aid robot is very far from the obstacles, so the obstacles stay in the **Free Layer**. At this moment, the component of the operators' intention force along the *x*-axis is greater than the repulsion force while the operator's intention force along the *y*-axis is close to zero. Thus, the robot is mainly controlled by the operators now. Meanwhile, the repulsion force is very small and mainly along the *y*-axis, as shown in Figures 2() and 3(). It is indicated that the walking-aid robot moves at a certain speed in the negative direction of the *y*-axis. After 10 seconds, the repulsion force obviously increases and the walking-aid robot gradually approaches the obstacle ob2. The obstacle is detected in the **Repulsion Interference Layer**. At the same time, the velocity of the robot along the *x*-axis decreases rapidly to zero, while the velocity of the robot along the *y*-axis increases with the repulsion force increasing. During this stage, the state of the walking-aid robot is “state 0.”

*Stage 2*. At *t* = 13s, the operators feel that the velocity of the walking-aid robot decreases; thus, the operators increase the thrust in the direction of the *x*-axis in order to make the walking-aid robot move again. At this moment, the obstacle ob2 is detected in the **Obstacle Emergency-Avoidance Layer**. According to the obstacle emergency-avoidance method, it meets the state-transition condition “a” then the state of the walking-aid robot changes from “state 0” to “state 1,” resulting that the velocity of the robot along the *x*-axis drops rapidly to zero and the robot moves at a fixed velocity along the *y*-axis. From Figures 2()(b) and 3()(b), we can see that the repulsion force remains constant. It indicates that in the “state 1,” the operators' intention force cannot affect the velocity of the robot in the stage 2 and the obstacles are not detected in the emergency obstacle avoidance region.

**Figure 12:**
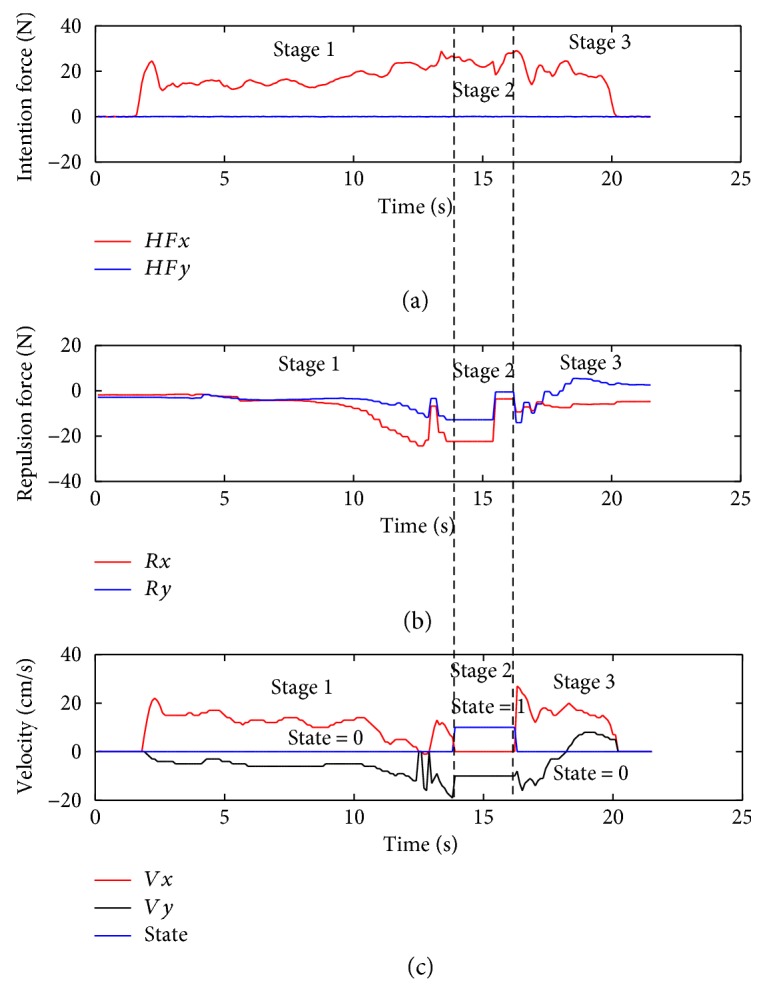
The experimental results with subject 1.

**Figure 13:**
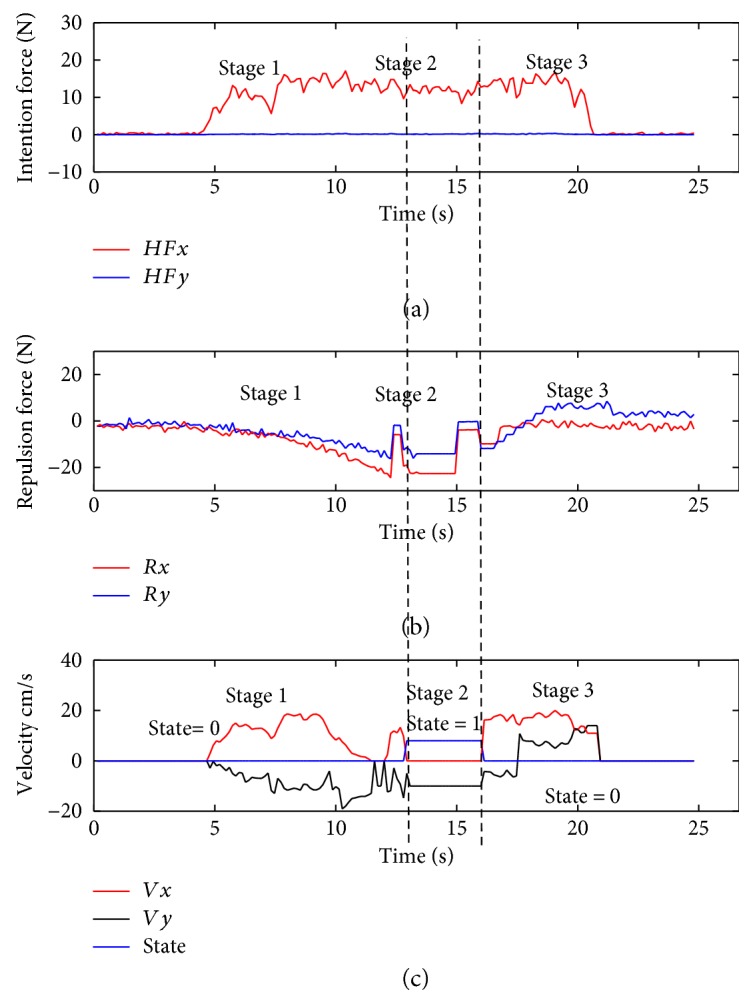
The experimental results with subject 2.

*Stage 3*. Due to the influence of the obstacle ob3, the walking-aid robot moves slowly along the positive direction of the *y*-axis according to the obstacle emergency-avoidance method. At *t* = 20s in Figure 12 and *t* = 21s in Figure 13, the operators stop applying the intention force to the walking-aid robot and the walking-aid robot stops moving.

**Figure 14:**
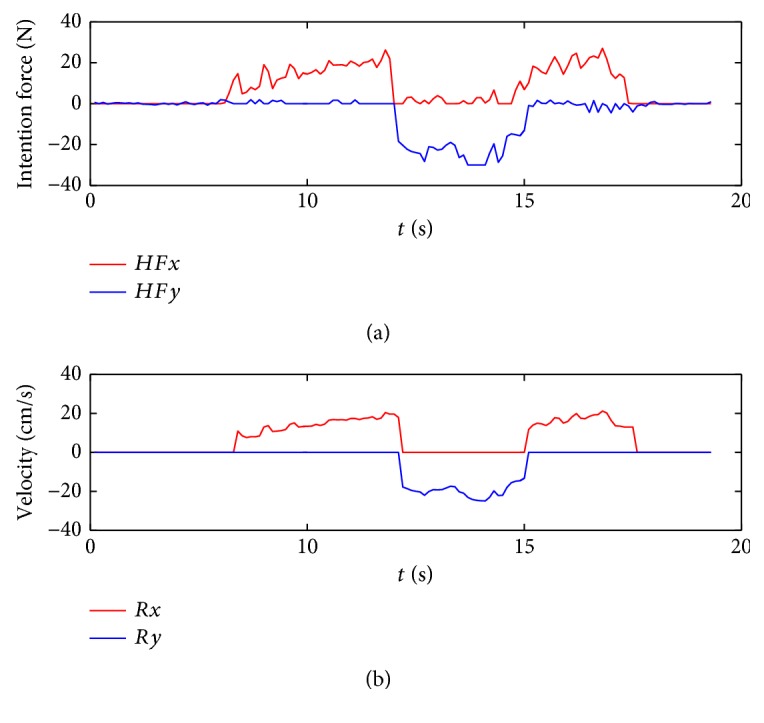
The experiment without shared control.

In the experiment with only the admittance control applied, it cannot reflect the effect of the obstacles in Figure 14. When the operator walks towards the obstacles, the operator have to avoid the obstacles on his own. Compared to the intention force *HFy* in Figures 12 and 13, the intention force *HFy* in Figures 12 and 13 is greater during the obstacle avoidance, which indicates that the shared-control method can save effort.

Over all, the human-robot interaction experiments show the effectiveness of the hierarchical shared control for the walking-aid robot. Based on the hierarchical shared-control algorithm, the robot can successfully help the operator to avoid obstacles and guide the operator to move in a feasible direction, which is really convenient.

## 5. Conclusions

In this work, we proposed a hierarchical control method for the walking-aid robot by combining the human motion intention recognition and the obstacle avoidance methods. It can save effort during obstacle avoidance and keeps the part of the operators' original walking intention. Using this control strategy, the walking-aid robot can autonomously choose different control algorithms to avoid obstacles based on the distance between the walking-aid robot and the obstacles. Also, the emergency obstacle avoidance mechanism is designed to ensure the security of the robot system. The experimental results show that the walking-aid robot can switch among different control algorithms smoothly and guide operators to walk safely.

## Figures and Tables

**Figure 10:**
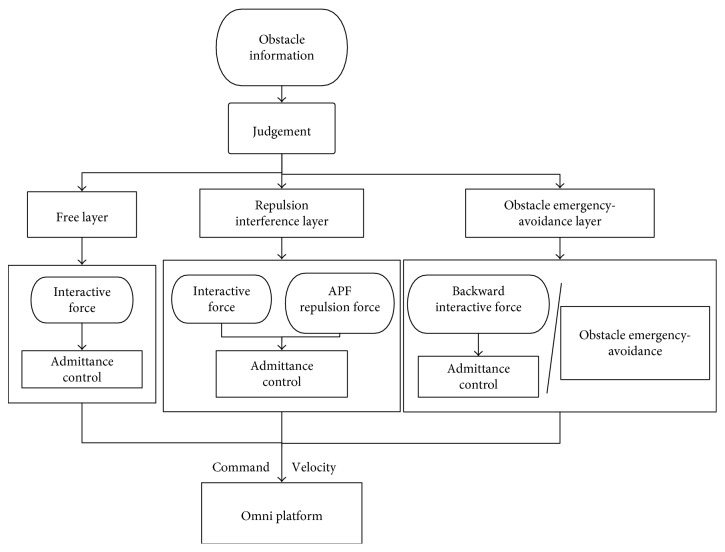
The hierarchical shared-control algorithm.

**Figure 11:**
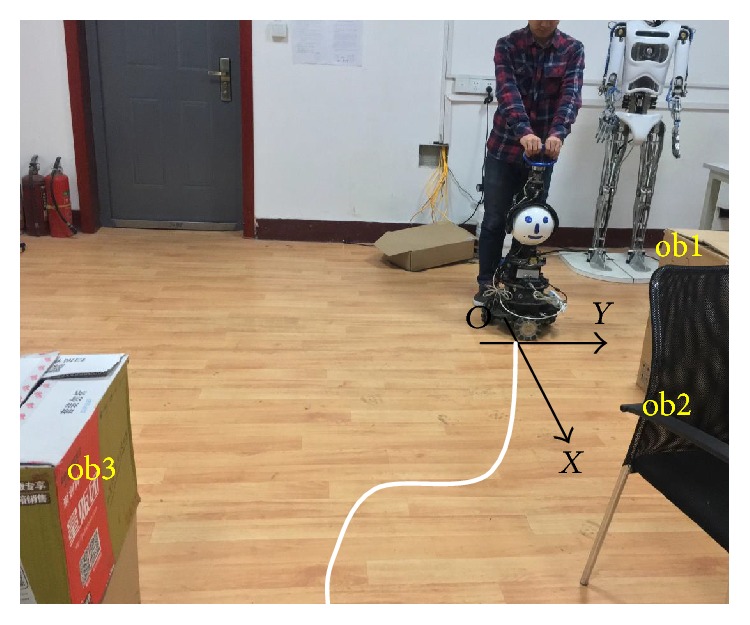
The human-robot interaction experiment.

**Algorithm 1:**
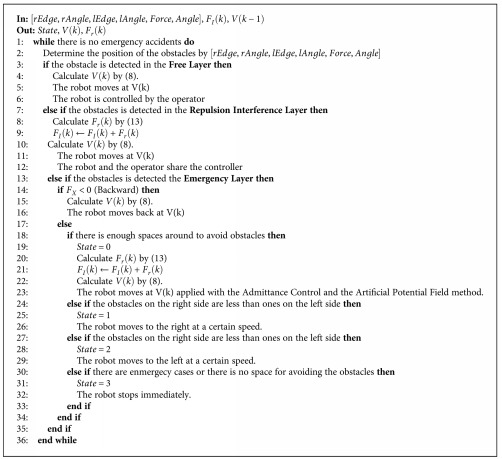
Hierarchical Shared Control.

**Table 1:** The states of the walking-aid robot.

State	Clarification	Action
0	No obstacles	Admittance control with APF
1	Obstacles are detected	Move to the right
2	Obstacles are detected	Move to the left
3	Emergency	Emergency stop

**Table 2:** The state-transition conditions.

State-transition condition	Clarification
a	The obstacles on the right side are less than the ones on the left side.
b	The obstacles on the left side are less than the ones on the right side.
c	There is enough space on the right side to avoid obstacles.
d	There is enough space on the left side to avoid obstacles.
e	There is no enough space on the right side to avoid obstacles.
f	There is no enough space on the left side to avoid obstacles.
g	The emergency cases or no obstacle-avoidance space.
h	The release of the emergency cases.

## References

[1] Kline K. A., Bowdish D. M. (2016). Infection in an aging population. *Current Opinion in Microbiology*.

[2] Hu C., Aeschlimann F., Chatzipirpiridis G. (2017). Spatiotemporally controlled electrodeposition of magnetically driven micromachines based on the inverse opal architecture. *Electrochemistry Communications*.

[3] Hu C., Vogler H., Aellen M. (2017). High precision, localized proton gradients and fluxes generated by a microelectrode device induce differential growth behaviors of pollen tubes. *Lab on a Chip*.

[4] Huang J., Tu X., He J. (2016). Design and evaluation of the RUPERT wearable upper extremity exoskeleton robot for clinical and in-home therapies. *IEEE Transactions on Systems, Man, and Cybernetics: Systems*.

[5] Kawamoto H., Kamibayashi K., Nakata Y. (2013). Pilot study of locomotion improvement using hybrid assistive limb in chronic stroke patients. *BMC Neurology*.

[6] Tanabe S., Saitoh E., Hirano S. (2013). Design of the Wearable Power-Assist Locomotor (WPAL) for paraplegic gait reconstruction. *Disability and Rehabilitation: Assistive Technology*.

[7] Kikuchi T., Tanaka T., Anzai K., Kawakami S., Hosaka M., Niino K. (2013). Evaluation of line-tracing controller of intelligently controllable walker. *Advanced Robotics*.

[8] Huang J., Guan Z. H., Matsuno T., Fukuda T., Sekiyama K. (2010). Sliding-mode velocity control of mobile-wheeled inverted-pendulum systems. *IEEE Transactions on Robotics*.

[9] Hirata Y., Hara A., Kosuge K. (2007). Motion control of passive intelligent walker using servo brakes. *IEEE Transactions on Robotics*.

[10] Wakita K., Huang J., Di P., Sekiyama K., Fukuda T. (2013). Human-walking-intention-based motion control of an omnidirectional-type cane robot. *IEEE/ASME Transactions on Mechatronics*.

[11] Khatib M., Chatila R. An extended potential field approach for mobile robot sensor-based motions.

[12] Iwatsuka K., Yamamoto K., Kato K. Development of a guide dog system for the blind with character recognition ability.

[13] Gonnot T., Saniie J. Integrated machine vision and communication system for blind navigation and guidance.

[14] Kaiser E. B., Lawo M. Wearable navigation system for the visually impaired and blind people.

[15] Kim C. J., Chwa D. (2015). Obstacle avoidance method for wheeled mobile robots using interval type-2 fuzzy neural network. *IEEE Transactions on Fuzzy Systems*.

[16] Fabrizio F., Luca A. D. (2017). Real-time computation of distance to dynamic obstacles with multiple depth sensors. *IEEE Robotics and Automation Letters*.

[17] Huang J., Di P., Fukuda T. Motion control of omni-directional type cane robot based on human intention.

[18] Di P., Hasegawa Y., Nakagawa S. (2016). Fall detection and prevention control using walking-aid cane robot. *IEEE/ASME Transactions on Mechatronics*.

[19] Yan Q. Y., Xu W. X., Huang J., Su P. C. Laser and force sensors based human motion intent estimation algorithm for walking-aid robot.

[20] Wang W., Hou Z. G., Cheng L. (2016). Toward patients’ motion intention recognition: dynamics modeling and identification of iLeg-An LLRR under motion constraints. *IEEE Transactions on Systems Man and Cybernetics Systems*.

[21] Khokar K., Alqasemi R., Sarkar S., Reed K., Dubey R. A novel telerobotic method for human-in-the-loop assisted grasping based on intention recognition.

[22] Han J. H., Lee S. J., Kim J. H. (2016). Behavior hierarchy-based affordance map for recognition of human intention and its application to human’ robot interaction. *IEEE Transactions on Human-Machine Systems*.

[23] Huang J., Huo W., Xu W., Mohammed S., Amirat Y. (2015). Control of upper-limb power-assist exoskeleton using a human-robot interface based on motion intention recognition. *IEEE Transactions on Automation Science and Engineering*.

[24] Sang H. C., Lee J. M., Kim S. J., Hwang Y., An J. Intention recognition method for sit-to-stand and stand-to-sit from electromyogram signals for overground lower-limb rehabilitation robots.

[25] Sheridan T. B. (1992). *Telerobotics, Automation and Human Supervisory Control*.

[26] Nudehi S. S., Mukherjee R., Ghodoussi M. (2005). A shared-control approach to haptic interface design for minimally invasive telesurgical training. *IEEE Transactions on Control Systems Technology*.

[27] Chen F., Di P., Huang J., Sasaki H., Fukuda T. Evolutionary artificial potential field method based manipulator path planning.

[28] Trieu H. T., Nguyen H. T., Willey K. Shared control strategies for obstacle avoidance tasks in an intelligent wheelchair.

[29] Huh W. G., Cho S. B. Optimal partial filters of EEG signals for shared control of vehicle.

[30] Xu W. X., Huang J., Wang Y. J., Tao C. J., Cheng L. (2015). Reinforcement learning-based shared control for walking-aid robot and its experimental verification. *Advanced Robotics*.

[31] Sathish S., Nithyakalyani K., Vinurajkumar S., Vijayalakshmi C., Sivaraman J. (2016). Control of robotic wheel chair using EMG signals for paralysed persons. *Indian Journal of Science and Technology*.

[32] Accogli A., Grazi L., Crea S. (2017). EMG-based detection of user’s intentions for human-machine shared control of an assistive upper-limb exoskeleton. *Wearable Robotics: Challenges and Trends*.

[33] Hong W., Tian Y. T., Dong Z., Zhou M. (2003). Extracting features from local environment for intelligent robot system. *Robot*.

